# Unraveling the impact of lysosomal dysfunction on myeloproliferative neoplasm

**DOI:** 10.1002/cam4.70238

**Published:** 2024-09-25

**Authors:** Hyundong Yoon, Dohoon Lee, Seulki Song, Bonil Koo, Jihyun Park, Tae Kon Kim, Sun Kim, Sheehyun Kim, Junshik Hong, Ja Min Byun, Dong‐Yeop Shin, Inho Kim, Youngil Koh, Sung‐Soo Yoon

**Affiliations:** ^1^ Cancer Research Institute, Seoul National University College of Medicine Seoul Republic of Korea; ^2^ Bioinformatics Institute, Seoul National University Seoul Republic of Korea; ^3^ BK21 FOUR Intelligence Computing Seoul National University Seoul Republic of Korea; ^4^ Interdisciplinary Program in Bioinformatics Seoul National University Seoul Republic of Korea; ^5^ Interdisciplinary Program in Artificial Intelligence Seoul National University Seoul Republic of Korea; ^6^ Division of Hematology/Oncology, Department of Medicine Vanderbilt University Medical Center Nashville Tennessee USA; ^7^ Department of Computer Science and Engineering Seoul National University Seoul Republic of Korea; ^8^ AIGENDRUG Co., Ltd Seoul Republic of Korea; ^9^ MOGAM Institute for Biomedical Research, Green Cross Corp Yongin Republic of Korea; ^10^ Department of Genomic Medicine Seoul National University Hospital Seoul Republic of Korea; ^11^ Center for Medical Innovation Seoul National University Hospital Seoul Republic of Korea; ^12^ Department of Internal Medicine Seoul National University Hospital Seoul Republic of Korea

**Keywords:** cytokine dysregulation, germline variant, lysosomal storage disease, myeloproliferative neoplasm, Oncostatin‐M

## Abstract

**Background:**

Lysosomal dysfunction (LD) impacts cytokine regulation, inflammation, and immune responses, influencing the development and progression of cancer. Inflammation is implicated in the pathogenesis of myeloproliferative neoplasm (MPN). With a hypothesis that LD significantly contributes to MPN carcinogenesis by inducing abnormal inflammation, our objective was to elucidate the pathophysiological mechanisms of MPN arising from an LD background.

**Methods:**

Genotyping of the LD background was performed in a cohort of MPN patients (n = 190) and healthy controls (n = 461). Logistic regression modeling, utilizing genotype data, was employed to estimate the correlation between LD and MPN. Whole transcriptome sequencing (WTS) (LD carriers = 8, non‐carriers = 6) and single‐cell RNA sequencing data (LD carriers = 2, non‐carriers = 2, healthy controls = 2) were generated and analyzed.

**Results:**

A higher variant frequency of LD was observed in MPN compared to healthy controls (healthy, 4.9%; MPN, 7.8%), with the highest frequency seen in polycythemia vera (PV) (odds ratio = 2.33, *p* = 0.03). WTS revealed that LD carriers exhibited upregulated inflammatory cytokine ligand–receptor genes, pathways, and network modules in MPNs compared to non‐carriers. At the single‐cell level, there was monocyte expansion and elevation of cytokine ligand–receptor interactions, inflammatory transcription factors, and network modules centered on monocytes. Notably, Oncostatin‐M (OSM) consistently emerged as a candidate molecule involved in the pathogenesis of LD‐related PV.

**Conclusions:**

In summary, an LD background is prevalent in MPN patients and leads to increased cytokine dysregulation and inflammation. OSM, as one of the potential molecules, plays a crucial role in PV pathogenesis by impairing lysosomal function.

## Abstract 2

Lysosomal dysfunction (LD) impacts cytokine regulation, inflammation, and immune responses, influencing the development and progression of cancer. Inflammation is implicated in the pathogenesis of myeloproliferative neoplasm (MPN). With a hypothesis that LD significantly contributes to MPN carcinogenesis by inducing abnormal inflammation, our objective was to elucidate the pathophysiological mechanisms of MPN arising from an LD background. Genotyping of the LD background was performed in a cohort of MPN patients (n = 190) and healthy controls (n = 461). Logistic regression modeling, utilizing genotype data, was employed to estimate the correlation between LD and MPN. Whole transcriptome sequencing (WTS) (LD carriers = 8, non‐carriers = 6) and single‐cell RNA‐sequencing data (LD carriers = 2, non‐carriers = 2, healthy controls = 2) were generated and analyzed. A higher variant frequency of LD was observed in MPN compared to healthy controls (healthy, 4.9%; MPN, 7.8%), with the highest frequency seen in polycythemia vera (PV) (odds ratio = 2.33, *p* = 0.03). WTS revealed that LD carriers exhibited upregulated inflammatory cytokine ligand‐receptor genes, pathways, and network modules in MPNs compared to non‐carriers. At the single‐cell level, there was monocyte expansion and elevation of cytokine ligand‐receptor interactions, inflammatory transcription factors, and network modules centered on monocytes. Notably, Oncostatin‐M (OSM) consistently emerged as a candidate molecule involved in the pathogenesis of LD‐related PV. In summary, an LD background is prevalent in MPN patients and leads to increased cytokine dysregulation and inflammation. OSM may play a crucial role in PV pathogenesis by impairing lysosomal function.

## 1 INTRODUCTION

Lysosomes are cellular organelles that play crucial roles in various biological processes, including macromolecular degradation, maintenance of homeostasis, cell adhesion and migration, pathogen destruction, plasma membrane repair, and apoptosis.^1^ Lysosomal storage disease (LSD), a collection of rare metabolic disorders, arises from mutations in genes responsible for regulating lysosomal homeostasis.^2^ These mutations evoke lysosomal dysfunction (LD) in genes encoding hydrolases, transporters, and enzyme activators, leading to the accumulation of macromolecules in the late endocytic system.^3^ This disturbance of lysosomal function can result in a wide range of dysregulated cytokines, abnormal inflammation, weakened immune response, endoplasmic reticulum stress, oxidative stress, degradation of the extracellular matrix, and impaired integrin‐β4‐mediated cell migration and invasion.^4^, ^5^ Especially, LD can foster the growth of pre‐malignant and malignant cells and may play a critical role in cancer development and progression, including invasion and metastasis.^6^, ^7^ These findings highlight the urgent need for further investigation into the impact of LD on diseases, particularly cancers.

Myeloproliferative neoplasms (MPN) are blood cancers characterized by the overproduction of specific blood cells or fibrous tissue in the bone marrow. The subtypes include primary myelofibrosis (PMF), polycythemia vera (PV), and essential thrombocythemia (ET).^8^ Mutations that primarily cause MPN in driver genes, such as *JAK2*, *MPL*, and *CALR* lead to the abnormal accumulation of hematopoietic stem cells (HSPCs) in the bone marrow,^9^, ^10^ even progressing to acute myeloid leukemia in some cases.^11^ Recent studies have suggested that chronic inflammation plays a vital role in the pathogenesis,^12^ impacting several aspects of the disease.^13^ As well delineated in JAK2‐mutated cases, chronic inflammation contributes to tumor clonal expansion by promoting the selective growth of tumor cells.^14^ Therefore, inflammation may be crucial in the pathogenesis of MPN and a potential therapeutic target.^14^, ^15^ Inflammation is a well‐known phenotype induced by LD.^5^ Macrophages in Gaucher disease impair autophagy function, which in turn activates *p65‐NFKB* and inflammasomes.^4^ This connection emphasizes the potential of targeting LD as an innovative therapeutic strategy in MPN management.

In this vein, a recent analysis of germline variants in 42 genes (Table S1) associated with LD, using pan‐cancer genomic data from the International Cancer Genome Consortium Pan‐cancer Analysis of Whole Genomes (PCAWG) project,^7^, ^16^ revealed the highest prevalence of LD‐related gene variants in MPNs. This significant discovery supports the proposed role of LD in driving MPN pathogenesis through the induction of abnormal inflammatory responses. Based on these insights, we hypothesize that LD is a critical driver of MPN carcinogenesis through its induction of abnormal inflammation. Consequently, our study aims to explore the potential pathophysiological link between LD and MPN to garner novel mechanistic insights. To achieve this, we have comprehensively genotyped patients with MPN and compared their profiles with healthy controls to validate previous findings and identify MPN subtypes most closely associated with LD. Additionally, we have examined immunological changes using both bulk and single‐cell RNA sequencing data for each MPN subtype in relation to their LD gene status. Through this approach, we aim to elucidate the contributions of individual cells within the tumor microenvironment, potentially unveiling new mechanistic insights and therapeutic targets.^17^ The outcomes of this research could pave the way for targeted therapies that mitigate the effects of LD in MPNs and potentially other related malignancies, offering new avenues for clinical intervention.

## 2 METHODS

### 2.1 Study cohort and participant selection

Between January 2020 and December 2022, a total of 190 patients (median age, 65 years; male‐to‐female ratio, 87:103) diagnosed with MPN at Seoul National University Hospital were prospectively enrolled, including 48 with PMF, 55 with PV, and 87 with ET. MPN diagnosis was established following the NCCN guidelines for the period from March 2022 to August 2022. Clinical data were derived from electronic and/or paper medical records. A cohort of 461 healthy donors (median age, 60 years) were prospectively recruited from Seoul National University Hospital Gangnam Healthcare Checkup Center as cancer‐free normal controls (CFNC). The CFNC subjects were aged >50 years and confirmed to be cancer‐free based on their healthcare checkup results. The CFNC cohort was described previously (https://www.ncbi.nlm.nih.gov/pmc/articles/PMC10580633/) (Table S2). This study was conducted with the approval of the Institutional Review Board (IRB) of Seoul National University Hospital (1908–170‐1059, 1705–031‐852). All procedures involving human participants were performed in strict accordance with the ethical standards of the IRB and with the 1964 Helsinki declaration and its later amendments or comparable ethical standards. Informed consent was obtained from all individual participants included in the study. All consent was written and documented in accordance with the guidelines prescribed by the World Medical Association Declaration of Helsinki.

### 2.2 Genotyping for lysosomal storage dysfunction

Genomic DNA was extracted from peripheral blood mononuclear cells (PBMCs) using the QIAamp DNA Tissue Kit (Heidelberg, Germany). The sequencing panel targeted 42 LD genes (Table S1). The library was prepared using the SWIFT Biosciences ACCEL‐NGS 2S DNA Library Kit (SWIFT Biosciences, Ann Arbor, MI, USA) and sequenced on the Illumina HiSeq 2500 platform. Sufficient coverage of variant filtering (Depth of coverage >10, Number of alternate alleles >5) and annotation were performed using ANNOVAR (version 2014 Apr 14)^18^ and VEPs (release‐96),^19^ respectively. Variants with a gnomAD (version 2.1.1)^20^ allele frequency of >0.5% were excluded. Tier‐1 variants include protein‐truncated variants (PTVs) and certain types of indels, whereas Tier‐2 variants are clinically significant variants defined in ClinVar (version 2019 June 18).^21^ Individuals were classified into two groups (LD rare variant carriers vs. non‐carriers) based on curated germline variant information (re S1).

### 2.3 Whole transcriptome sequencing (WTS), data generation, and analysis

#### 2.3.1 Sample selection

Bulk RNA‐sequencing data were generated from 14 patients diagnosed with MPN. Using the LD genotype, we selected eight LD carriers (PMF = 2, PV = 3, and ET = 3) and eight non‐carriers (PMF = 1, PV = 2, and ET = 3) with available RNA from bone marrow samples (Table S3, Figure S2A).

#### 2.3.2 WTS data sequencing

For the WTS data, a subset of patients with MPN was selected (LD carriers = 8, non‐carriers = 6). Total RNA was isolated from PBMCs samples, and RNA‐sequencing libraries were prepared using the TruSeq‐stranded mRNA kit (Illumina, San Diego, CA, USA) from Illumina. Libraries were sequenced following the manufacturer's protocol on an Illumina Novaseq 6000 platform. The quality of cDNA libraries was evaluated using an Agilent 2100 Bio Analyzer (Agilent Technologies, Santa Clara, CA, USA).

#### 2.3.3 Whole transcriptome data analysis

The whole transcriptome data were aligned to the human reference genome (version Hg38).^22^ Expression abundances were inferred, and read count values were obtained for each gene^23^ based on the mapping results. The transcriptomic data (n = 14) generated in this study were divided into LD carriers and non‐carriers based on the presence of LD germline variants.

#### 2.3.4 Differentially expressed genes

For the WTS data, normalization was performed, and genes with zero raw mRNA counts were removed. Differentially expressed genes (DEGs) analysis was conducted.^24^ Genes satisfying the Log2FC >0.5. <−0.5 and *p* < 0.05 were classified as significantly upregulated or downregulated genes.

#### 2.3.5 Classification of cytokine ligand–receptor (LR)genes

To identify LR genes among statistically significant DEGs, we utilized a database of human LR interactions.^25^ Cytokine LR genes were classified based on gene information from the Human Protein Atlas database (proteinatlas.org).^26^

#### 2.3.6 Gene set enrichment analysis (GSEA) using transcriptomic data

GSEA utilized the Hall marker (H), curated (C2), and ontology (C5) gene set databases from MsigDB.^27^ Calculated normalized enrichment scores (NES) and statistically significant upregulated or downregulated pathways were classified (NES >0, < 0, and *p* < 0.05) based on the NES criteria.^28^

#### 2.3.7 Differential correlation network analysis

Pearson's correlation coefficients were calculated using gene expression values, and correlation network edges were generated using a threshold of >0.6. Only edges present in the LD carrier group were retained. Differential network edges were built using the STRING template network^29^ with a reliability score of >700. Cytoscape^30^ was used for visualization. Functional pathway enrichment analysis^31^ was conducted using the Gene Ontology Biological Process and TRRUST databases.^32^

#### 2.3.8 Transcription factor enrichment analysis using transcriptomic data

The transcription factor (TF) database^33^ was used to infer differential TF activity levels using potential target genes and the NES.^34^ TFs were classified as significantly upregulated or downregulated based on the NES criteria and a *p*‐adjusted value threshold (NES >0, < 0, and *p*‐adjusted value <0.05).

### 2.4 Single cell RNA data generation and analysis

#### 2.4.1 Sample selection

Using genotype data, two LD carriers with PV and two LD non‐carriers with PV who had available RNA samples from bone marrow were selected for single‐cell RNA (scRNA) data generation (Table S4).

#### 2.4.2 Single‐cell RNA data sequencing

RNA was isolated from bone marrow mononuclear cells, and the chromium single‐cell 3 prime‐GEM, Library & Gel Bead kit v3 from 10X Genomics (San Francisco, Ca, USA) was used for library preparation. Cells were mixed with the reverse master mix and loaded into chip channels to capture 500–10,000 individual cell transcriptomes. GEMs were generated using a Chromium Controller (10X Genomics). The libraries were sequenced on an Illumina HiSeq X Ten platform, generating read pairs with a minimum of 10,000 cells per sample. scRNA data from healthy donors were obtained from a published dataset^35^ (GSE120221), and two high‐quality samples (GSM3396167 and GSM3396169) were selected and used from 20 publicly available samples (Table S4).

#### 2.4.3 Single‐cell RNA data preprocessing

scRNA‐seq data were aligned using Cell Ranger^29^ to generate a raw gene‐cell count matrix. Quality control removed noise and low‐quality cells,^30^, ^31^ and outliers were eliminated based on principal component analysis (PCA) and specific thresholds (nUMI, nFeature, and Mitochondria expression). Normalization,^32^ variable gene selection, SNN graph construction, and UMAP^27^ visualization were performed. Red blood cell clusters were excluded in one sample (MPN 4814). Integration addressed batch effects,^33^ and cell types were identified using DEGs and marker genes^34^ (Method S2).

#### 2.4.4 Single‐cell DEGs

For scRNA, the MAST^36^ (ver. 1.24.1) framework in the Seurat^37^ package was utilized, and the FindMarkers function was applied for DEG analysis (avg_log2FC >0.25, <−0.25, and *p* < 0.05) and classified as differentially upregulated or downregulated genes.

#### 2.4.5 Comparison of differences in cell type proportion

Cell type distributions were compared by calculating percentages, performing permutation tests, and bootstrapping. Statistical differences were determined based on the Log2 fold distribution and FDR^38^ (Log2FD >0.3, < −0.3, and *p*‐adj. value <0.05).

#### 2.4.6 Cell‐to‐cell interaction inference

To measure LR gene interactions, those expressed in at least 5% of the cell types and belonging to the secreted signaling category were considered.^25^ Differences in interaction strength between groups were assessed based on the communication probability strength. The results were validated using pseudo‐bulk DEG analysis.

#### 2.4.7 Pseudo‐bulk DEG analysis

A pseudo‐bulk matrix was generated from the gene‐by‐cell matrix, and differential expression analysis was performed.^39^ Genes exhibiting significant upregulation or downregulation were classified based on LogFC (>0.25, < −0.25) and *p* (<0.05).

#### 2.4.8 Single‐cell co‐expressed correlation network analysis

Pearson correlation coefficients were calculated^40^ for the integrated scRNA data. Correlation networks were constructed for specific cell types, and differentially expressed network modules were identified and validated using enrichment analysis.^31^

#### 2.4.9 Gene regulatory network (GRN) analysis

A GRN was constructed based on gene correlation values.^41^ TF‐binding targets were analyzed, and TF activity scores were calculated.^42^

### 2.5 Statistical analysis

The chi‐square test was used to compare LD genotype prevalence across the disease and CFNC groups. A logistic regression model was used to calculate the odds ratio OR to determine the risk of LD germline variants in patients with cancer and healthy controls. A one‐sided t‐test (*p* < 0.05) was used, and all analyses were conducted in the R programming environment (http://www.r‐project.org).

## 3 RESULTS

### 3.1 Genotyping of patients with MPN and cancer‐free healthy controls

Through germline variant analysis (Method S1), we determined that an LD‐carrier genotype is present when there are either Tier‐1 variants, which include protein‐truncating variants (PTVs) and specific types of insertions/deletions, or Tier‐2 variants that are clinically significant as defined in ClinVar (version June 18, 2019) among the 42 genes essential for the primary functions of lysosomes (Table S1). As a result of genotyping the 42 LD‐related genes in patients with MPN (N = 190) and CFNC cohorts (n = 461), potentially pathogenic germline single nucleotide variants, and/or insertion/deletions (indels) in 11 genes (*ASAH1*, *GALC*, *GBA*, *GNPTAB*, *HEXB*, *HYAL1*, *MAN2B1*, *SGSH*, *GAA*, *GLB1*, and *TPP1*) were identified. Among the 39 identified germline variants, 29 were Tier 2 variants, which contained pathogenic information from Clinvar. There were 15 Tier 1 PTVs (five variants were categorized as both Tier 1 and Tier 2; Figure S1A; Table S5). Among the 190 patients with MPN, 8% had one or more LD germline mutations. When examining the frequency of LD variants by MPN subtype, PMF, PV, and ET had a frequency of 8%, 11%, and 6%, respectively. In contrast, the CFNC group harbored LD germline mutations at a frequency of 5% (Figure S1B, Tables S2 and S5). Logistic regression analysis revealed that PV has a statistically significant outcome, with and OR of 2.33 (*p* = 0.03) (Figure S1C). These findings align with those of a previous study^16^ and have directed our focus toward PV for subsequent mechanistic studies using transcriptome data.

### 3.2 Differentially expressed LR genes at the whole transcriptome level

**FIGURE 1:**
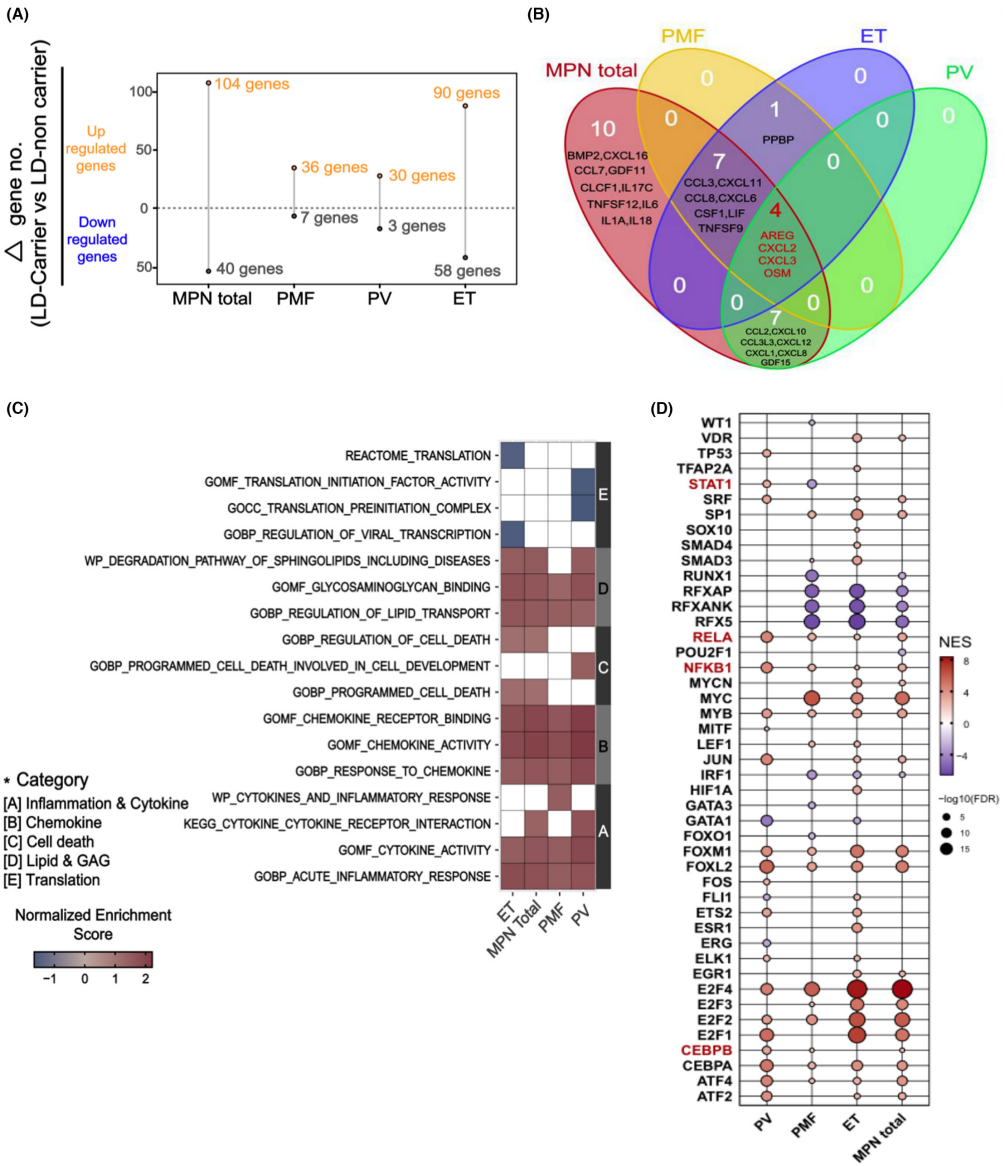
Regulation changes of inflammatory cytokine LR genes, pathways, and transcription factors at the bulk RNA level. (A) Number of upregulated and downregulated LR genes in MPN total and subtype diseases with an LD background. (B) Venn diagram depicting overlapping inflammatory cytokine LR genes in each group with an LD background. (C) Pathways that are upregulated or downregulated in each group with an LD background. (D) Transcription factors that are upregulated or downregulated in each group with an LD background (highlighted in red represent inflammatory transcription factors). LD, lysosomal dysfunction; LR, ligand‐receptor; MPN, myeloproliferative neoplasm.

A number of LR genes were identified in the MPN group, followed by the ET, PMF, and PV subtypes (Figure 1A). Notably, among the LR genes, a substantial proportion encoded inflammatory cytokines. Overlapping genes were observed in all groups, and *OSM*, *CXCL2*, *CXCL3*, and *AREG* were upregulated in LD carriers compared with those in non‐carriers (Figure 1B).

### 3.3 Pathway and TF analysis using WTS data

GSEA revealed downregulation of lysosomal function‐related genes in all MPN and disease subtypes (PMF and PV) in LD carriers. In contrast, pathways associated with glycosaminoglycan (GAGs) and sphingolipid/lipid metabolism were upregulated in LD carriers across all MPN and all disease subtypes compared with that in non‐carriers, suggesting their accumulation owing to impaired lysosomal enzyme function. Additionally, pathways involved in cell death and stress were upregulated in total MPN and in disease subtypes (PV and ET) (Figure 1C). Immune‐ and inflammation‐related pathways were upregulated in total MPN, and all disease subtypes aligned with the previous DEG analysis (Figure 1C).

The activity of various TF's was differentially regulated between LD carriers and non‐carriers. Notably, TF's involved in inflammation, such as *NFKB1*, *RELA*, and *CEBPB*, were upregulated. When MPN subtypes were considered, *NFKB1* and *RELA* were identified as overlapping upregulated TFs in the total MPN group and all subtypes (PMF, PV, and ET). *CEBPB* showed overlapping overexpression in PV and PMF (Figure 1D).

### 3.4 Differential correlation network at the whole transcriptome level

**FIGURE 2:**
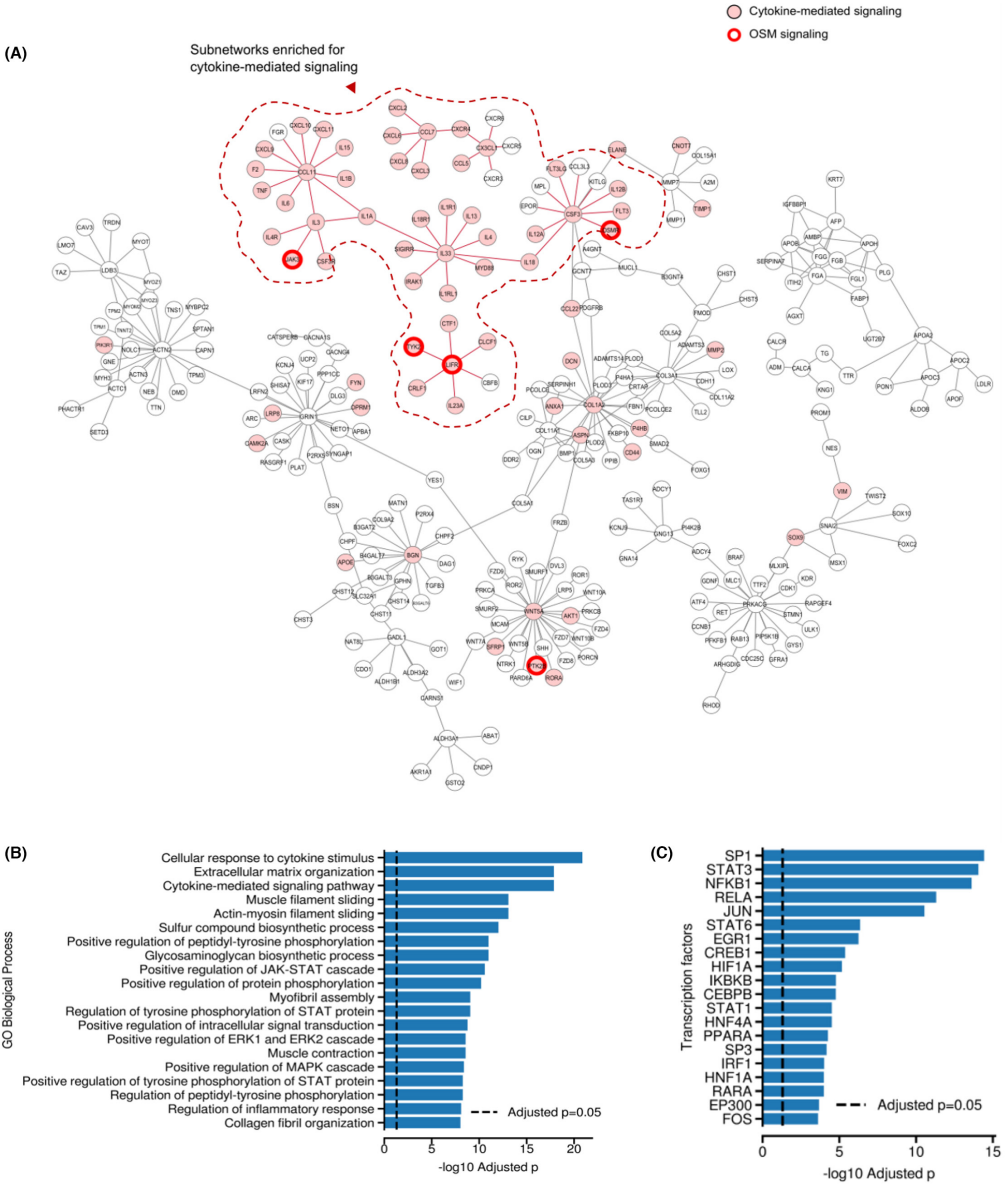
Differential gene correlation network and regulated pathways and transcription factors in the LD background. (A) Differential gene correlation network observed in the LD background group (cytokine‐mediated and OSM signaling highlighted in red). (B) Enhanced pathways in the differential network. (C) Enriched transcription factors in the differential network. LD, lysosomal dysfunction; OSM, Oncostatin‐M.

Using a gene correlation network, we observed a differential network consisting of 709 edges and 822 genes that appeared exclusively in the LD carriers (Figure 2A). Enrichment analysis revealed that this network was associated with cytokine‐mediated signaling pathways, including the signaling pathways of differentially expressed genes in *OSM* (Figure 2B), which were previously identified in duplicate. Moreover, we observed that the inflammatory TFs (*NFKB1*, *RELA*, *STAT1*, and *CEBPB*) were differentially co‐expressed regulators (Figure 2C), which is consistent with the previous analysis.

### 3.5 Single‐cell transcriptomic analysis of PV patients according to LD carrier status

**FIGURE 3:**
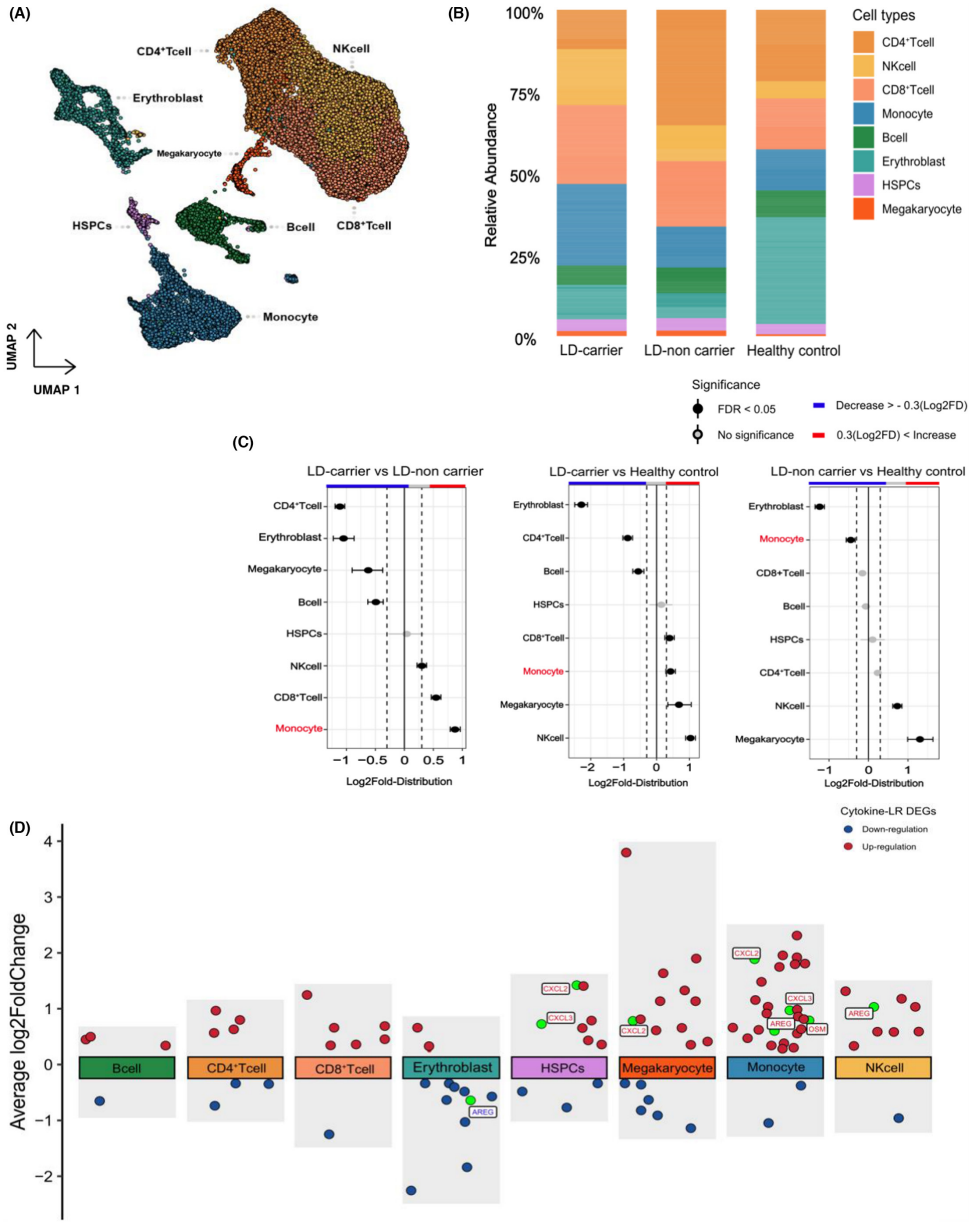
Cell proportion and differential expression cytokine LR genes in single‐cell transcriptome data. (A) UMAP visualization of single‐cell RNA from integrated patients with PV (*n* = 4; two LD carriers and two non‐carriers) and healthy donors (*n* = 2). (B) Relative cell population across groups. (C) Statistical testing result of differential cell population between groups. (D) Differential cytokine LR gene expression across the entire cell type between groups with and without LD background, with cytokine LR genes (Target Cytokine LR genes were previously duplicated in the bulk RNA data labeled). LD, lysosomal dysfunction; LR, ligand–receptor; PV, polycythemia vera; UMAP, Uniform Manifold Approximation and Projection.

We analyzed scRNA data from patients with PV (n = 4; two LD carriers and two non‐carriers) and healthy donors (n = 2) (Figure 3A, Figure S3A–C). The cell types were identified using canonical marker genes (Figure S2A), and eight cell types were identified (Figure 3A). Differences in cell type proportions between groups were quantified and compared (Figure 3B). We observed that CD8^+^ T‐cell and monocyte populations were significantly increased in LD carriers compared with that of non‐carriers (Figure 3C). Moreover, CD8^+^ T cells and monocytes were significantly increased in LD carriers compared with that of LD non‐carriers (Figure 3C).

#### 3.5.1 Differentially expressed cytokine LR genes at the single‐cell level

**FIGURE 4:**
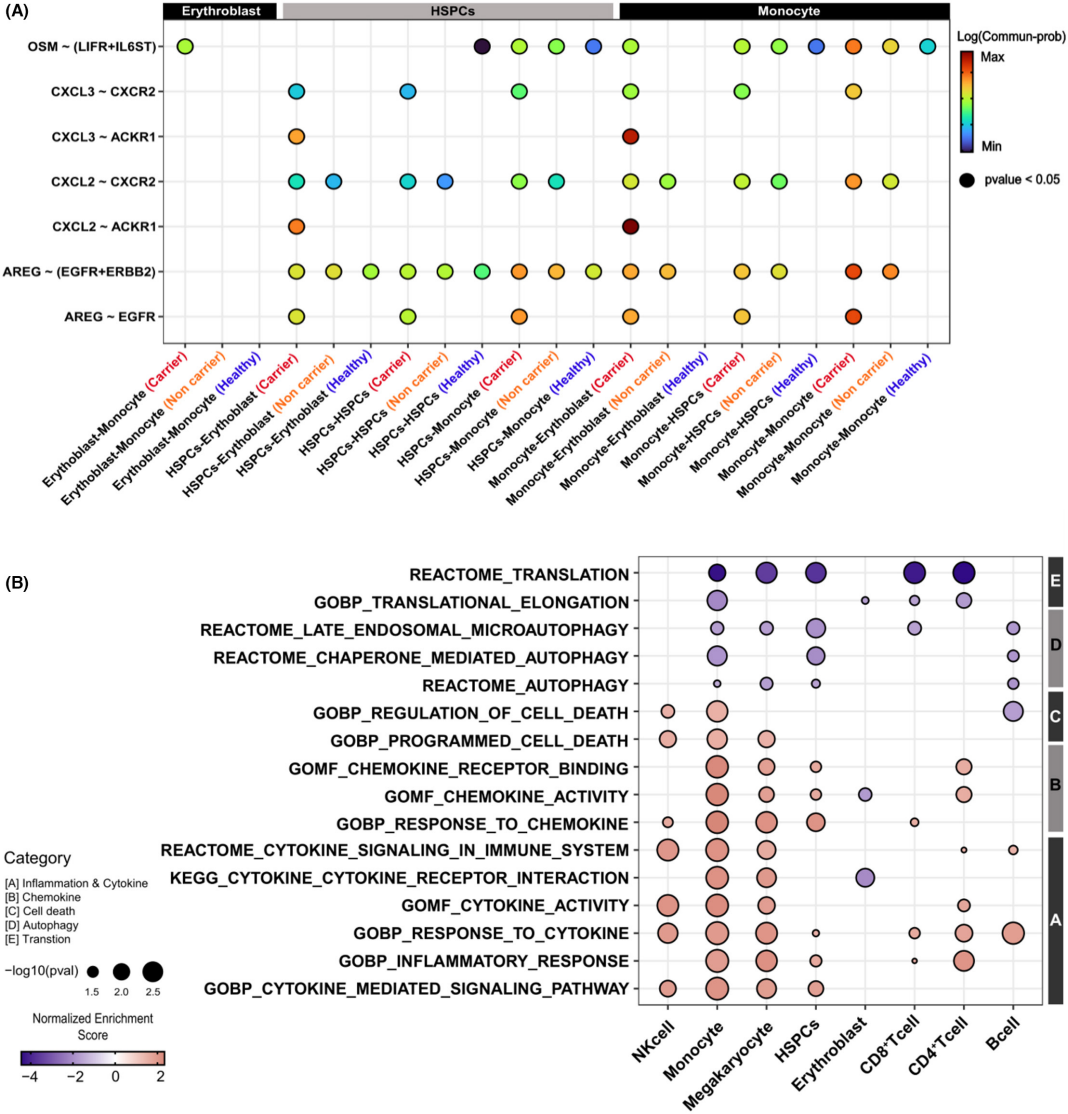
Regulated intracellular interaction and pathways in single‐cell transcriptome data. (A) Comparison of intercellular interaction strength of duplicated target cytokine LR genes in target cell types within each group. (B) Pathways regulated by patients with LD background across the entire cell type compared between patients with and without LD background. LD, lysosomal dysfunction; LR, ligand–receptor.

We identified differentially expressed cytokine LR genes (Figure S4A,B) with the largest DEGs observed in monocytes. A number of cytokine LR genes were preferentially expressed in LD carriers across all cell types, with the highest expression observed in monocytes (Figure 3D). Among these genes, *OSM* was specifically highly expressed in the LD carriers. GSEA of scRNA indicated that pathways related to cytokines, inflammation, chemokines, and apoptosis were upregulated in PV (both LD carriers and LD non‐carriers) compared with that in healthy donors. Conversely, pathways related to biogenesis, translation, and autophagy were downregulated in the LD carriers (Figure 4B). Further analysis of PV samples according to the LD carrier status revealed that LD‐carrier cells exhibited stronger upregulation and downregulation of these pathways than LD‐non‐carrier cells. Again, this phenomenon was most pronounced in the monocytes.

#### 3.5.2 Cell‐to‐cell interactions

To investigate the intercellular interactions of the cytokine LR genes in the WTS data (Figure 1B), we focused on erythroblasts and HSPCs, which are the primary differentiating cells of cancer cells in the PV and the immune cells of monocyte.^43^ Cytokine LR genes, including *OSM*, *CXCL2*, *CXCL3*, and *AREG*, which were duplicated in all cases in the previous WTS data analysis, exhibited autocrine or paracrine interactions between monocytes and erythroblasts or HSPCs (Figure 4A). These interactions were stronger in patients with PV (both LD carriers and non‐carriers) than in healthy donors (Figure S5A,B). The results were validated using pseudo‐bulk DEG analysis (Figure S5A–C). The interaction was stronger in LD carriers than in non‐carriers. Notably, *OSM* interaction was stronger in LD carriers than in non‐carriers (Figure 4A, Figure S5C).

#### 3.5.3 Differential correlation network modules in monocytes

**FIGURE 5:**
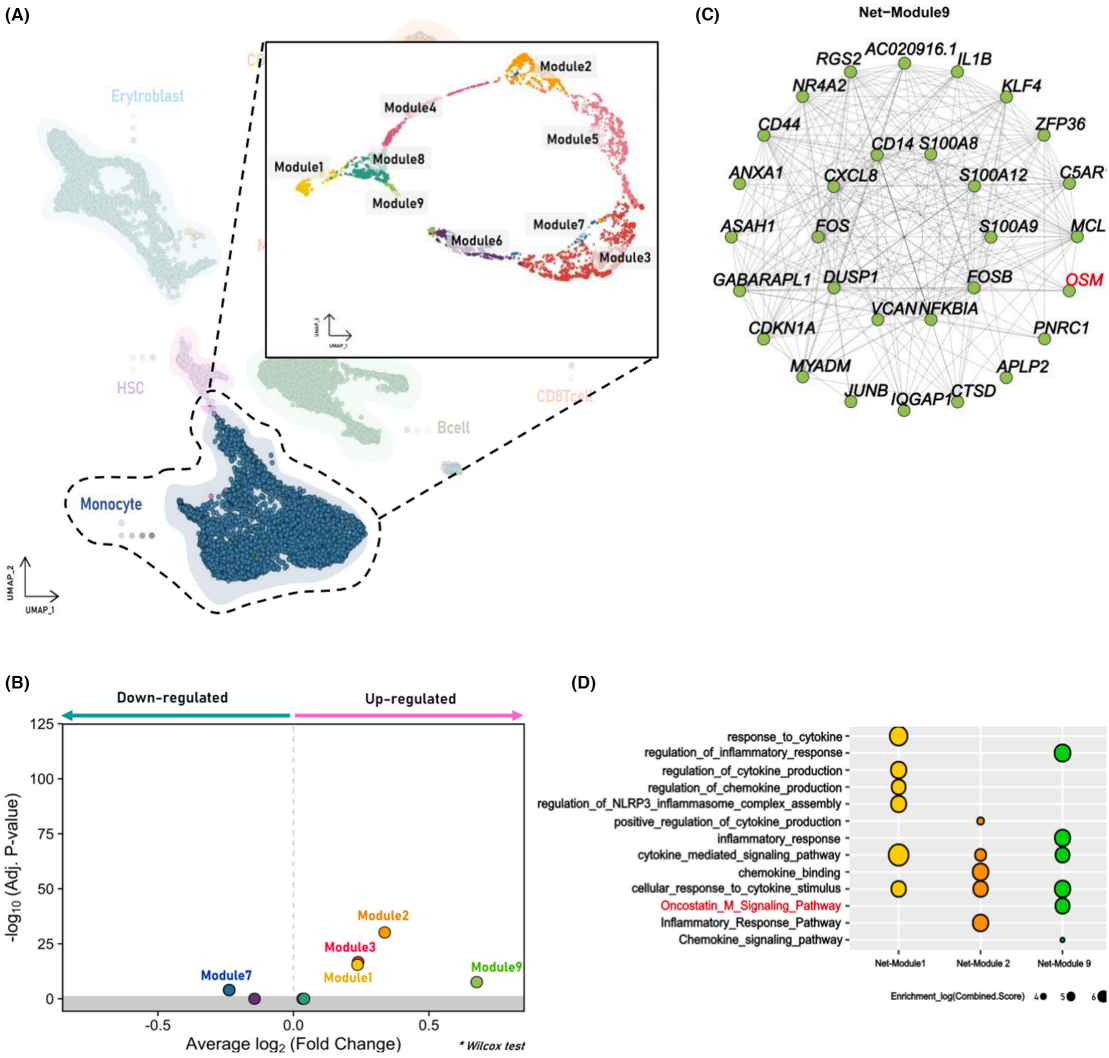
Identified regulated network modules and hub genes in the differential gene correlation network of monocytes and enriched network pathways. (A) Visualization of the gene correlation network modules identified in monocytes using UMAP. (B) Volcano plot illustrating the differentially regulated network modules in monocytes. (C) Hub genes comprise network module 9, upregulated in monocytes (OSM highlighted in red). (D) Enriched pathways associated with the upregulated network module. OSM, Oncostatin‐M; UMAP, Uniform Manifold Approximation and Projection.

A correlation network analysis helped identify nine network modules (Figure 5A), with five modules meeting the *p*‐adjusted value of <0.05, as adjusted by the Wilcoxon test (Figure 5B): Modules 1, 2, 3, and 9 were upregulated, whereas module 7 was downregulated. Pathway enrichment analysis of the upregulated network modules revealed the enrichment of cytokine, chemokine, and inflammation‐related pathways (Figure 5D). In particular, for module nine, by measuring the ranking of all hub genes, *OSM* ranked among the top 30 hub genes in the network module (Figure 5C). Accordingly, the *OSM* signaling pathway was enriched (Figure 5D).

#### 3.5.4 Investigation of OSM downstream transcription factors

**FIGURE 6:**
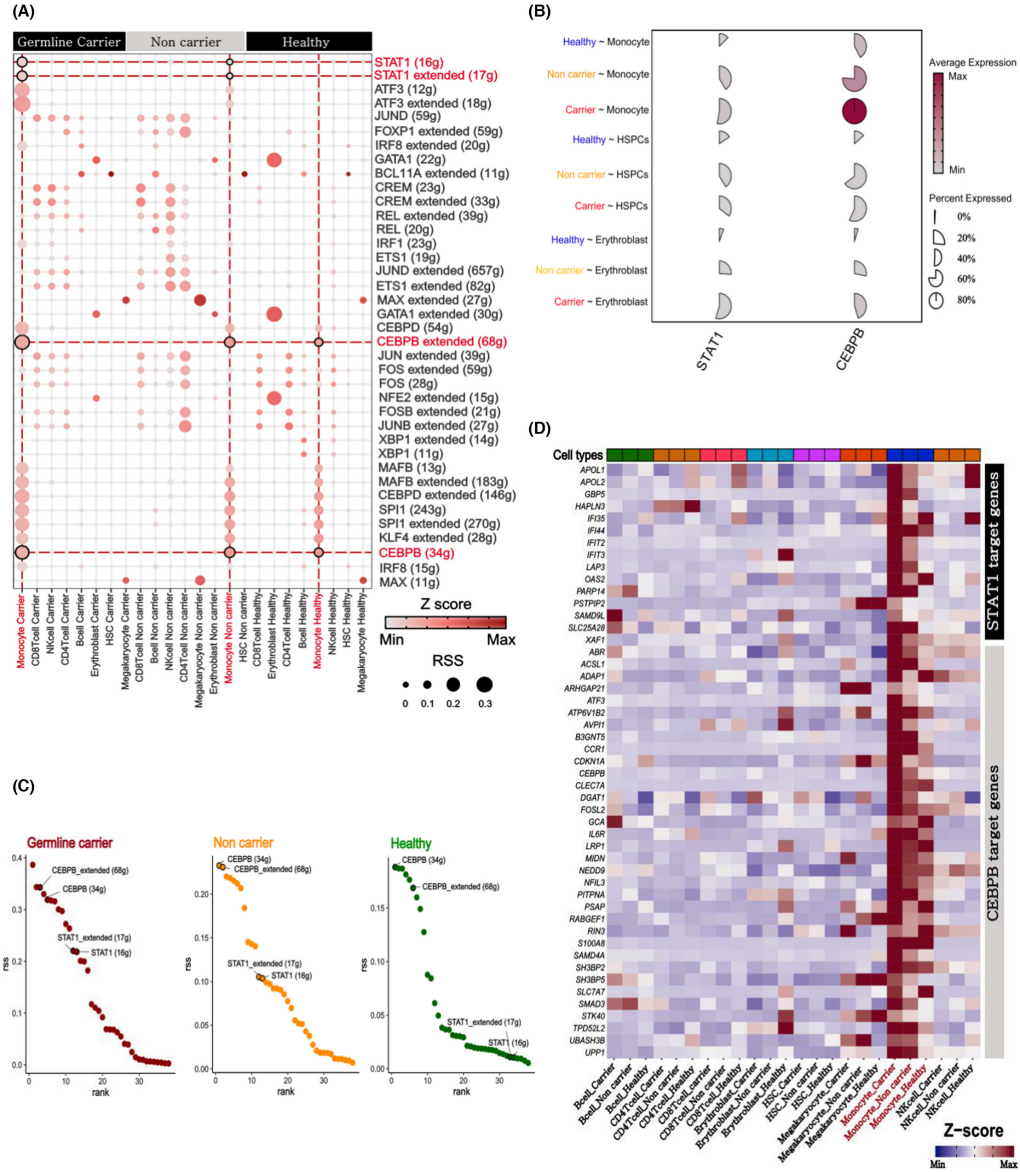
Gene regulatory network (GRN) at the single‐cell level. (A) Transcription factor activity scores in each cell group (inflammatory target transcription factors highlighted in red). (B) Comparison of target transcription factor expression at the single‐cell level (healthy cells highlighted in blue, PV patients without LD background highlighted in yellow, PV patients with LD background highlighted in red). (C) Ranking of transcription factor activity in each group (labeled with target transcription factors). (D) Comparison of downstream target gene expression values of target transcription factors in each group (highlighted in red for expression in monocytes in each group). LD, lysosomal dysfunction; PV, polycythemia vera.

OSM plays a central role in cytokine production and regulation,^44^ and directly intervenes in chronic inflammation. STAT1, a downstream inflammatory factor of OSM in the cell nucleus, demonstrated higher activity and ranking in both LD carriers and non‐carriers. GRN analysis reveal that LD carriers exhibited higher scores and rankings, whereas healthy donors displayed lower STAT1 activity and rankings (Figure 6A, C). Another downstream inflammatory inducer of OSM, CEBPB, exhibited activity in LD carriers, non‐carriers, and healthy donors, with LD carriers displaying the highest activation scores (Figure 6A). We compared the expression of the downstream target genes, STAT1 and CEBPB, which revealed that in LD carriers and non‐carriers, target gene expression was higher than that in healthy donors, with LD carriers having the highest expression of target genes (Figure 6D). These findings were further substantiated by comparing the expression levels of genes encoding each TF (Figure 6B).

## 4 DISCUSSION

Our results reveal a higher frequency of LD germline variants in the overall MPN population through germline variant analysis. Within disease subgroups, a statistically significant enrichment of LD germline variants was observed in polycythemia vera (PV). Compared to previously published research, these findings align with previous results from the PCAWG dataset,^16^ providing validation for a robust association between dysregulation of lysosomal function caused by LD genetic variants and MPN carcinogenesis in a distinct ethnic group (Korean). Moreover, despite being categorized under the same MPN umbrella, emerging evidence suggests nuanced differences in the pathophysiology of essential thrombocythemia (ET), polycythemia vera (PV), and primary myelofibrosis (PMF).^45^ Therefore, it is particularly meaningful that we identified a specific entity, PV, showing the strongest correlation with lysosomal dysfunction.

After establishing an association, it becomes imperative to ascertain whether the connection is a mere bystander effect or indicative of a causal relationship. To address this, understanding alterations in functional patterns based on the LD germline variant status using both bulk and single‐cell RNA data is crucial. At the bulk RNA level, we observed a reduction in functions directly linked to lysosomes in the presence of the variant, implying the upregulation of accumulated glycosaminoglycans (GAGs), sphingolipids, and lipid‐related pathways. Additionally, an elevation in functions associated with cell death, stress, immunity, and inflammation was noted in cases with LD variants. Notably, the consistent upregulation of inflammatory cytokine LR genes, transcription factors, and networks linked to immune and inflammatory functions suggests the impact of impaired lysosomal enzyme function. Our observation coincides with the established knowledge that the impairment of host cells encompassing autophagy with self‐degradation due to lysosomal dysfunction mediated mechanisms exerts a widespread and substantial impact on immune responses and inflammation.^5^ At the single‐cell level, utilizing single‐cell RNA‐sequencing data from PV patients, we observed a general decrease in lysosome‐related functions and an upregulation of lipid‐related pathways across the identified cells. This evidence aligns with the impaired lysosomal enzyme function identified at the bulk RNA level. It is of note that dysregulation in lysosomal function aligns with the significance of lipid metabolism, influenced by LD, in maintaining homeostasis and regulating autophagy.^46^ Specifically, there is a well‐established link between defective autophagy due to LD‐mediated lipid metabolism and the activation of inflammatory responses.^47^ Within immune cells, there is an overall upregulation of LR genes associated with inflammatory cytokines, particularly in monocytes. These findings were further substantiated by cell–cell interaction analysis in monocytes, revealing a higher interaction intensity of LR genes for inflammatory cytokines compared to the control group (LD non‐carrier, healthy donor). Monocytes express a variety of receptors that detect microenvironmental changes. Due to their high flexibility, heterogeneity, and responsiveness to environmental stimuli, monocytes serve as indicators of various inflammatory conditions and play a crucial role in generating inflammatory cytokines. Therefore, the significance of monocytes in inflammation supports the rationale behind our results.^4^ All in all, this suggests that impaired lysosomal enzyme function may exacerbate the inflammatory environment.

Significantly, at the molecular level, among the various LR genes identified for inflammatory cytokines, we focused on OSM, a key player in cytokine production and regulation,^44^ as the target ligand for downstream signaling. Consequently, crucial transcription factors (TFs), CEBPB, and STAT1, directly associated with OSM in gene regulatory networks, exhibited higher activity scores in monocytes carrying LD germline variants. This observation was further substantiated by the differential expression of genes encoding these TFs. Moreover, the expression patterns of the target genes for these transcription factors and their expression levels were elevated in individuals carrying LD germline variants. OSM, part of the IL‐6 cytokine family, is widely recognized for its engagement in various biological processes and cellular responses, such as growth, differentiation, and inflammation. Our research findings show that OSM's interaction intensifies the inflammatory environment by activating crucial transcription factors and their target genes. As suggested, this underscores OSM's pivotal role in amplifying inflammatory conditions and associated genes.^44^ However, this study has several limitations. First, the WTS data did not include samples from healthy donors, which makes it difficult to compare lysosomal function at the whole transcriptome level with that of healthy individuals. Secondly, the scRNA data were generated solely from PV patients, leaving it unclear whether other MPN disease subtypes (PMF, ET) also contribute to cytokine dysregulation and an inflammatory environment, particularly in monocytes. Finally, we did not conduct experimental studies to investigate the mechanisms underlying our findings. Therefore, future studies will aim to generate additional data and experimentally validate our results to overcome these limitations.

In summary, we newly discovered a strong association between LD germline variants and MPN. In particular, these variants may be associated with cytokine dysregulation and induction of an inflammatory environment, as observed in our transcriptomic data. Furthermore, in the scRNA data of patients with PV, this phenomenon was mainly concentrated in monocytes, and we observed that *OSM* was highly expressed in monocytes carrying the genetic variant. These findings suggest that *OSM* could be a pivotal target molecule for future longitudinal studies on patients with PV‐carrying LD germline variants.

## AUTHOR CONTRIBUTIONS

**Hyundong Yoon:** Conceptualization (lead); data curation (lead); formal analysis (lead); investigation (lead); methodology (lead); validation (lead); visualization (lead); writing – original draft (lead); writing – review and editing (lead). **Dohoon Lee:** Conceptualization (equal); data curation (equal); formal analysis (lead); methodology (lead); visualization (lead); writing – original draft (equal); writing – review and editing (equal). **Seulki Song:** Formal analysis (equal); methodology (equal); writing – review and editing (supporting). **Bonil Koo:** Formal analysis (equal); methodology (equal); writing – review and editing (supporting). **Jihyun Park:** Conceptualization (supporting); validation (equal); writing – review and editing (equal). **Tae Kon Kim:** Conceptualization (supporting); supervision (supporting); writing – review and editing (equal). **Sun Kim:** Conceptualization (supporting); supervision (equal); writing – review and editing (equal). **Sheehyun Kim:** Data curation (supporting); writing – review and editing (supporting). **Junshik Hong:** Conceptualization (equal); data curation (supporting); supervision (supporting); writing – review and editing (equal). **Ja Min Byun:** Conceptualization (equal); supervision (supporting); writing – review and editing (equal). **Dong‐Yeop Shin:** Conceptualization (equal); supervision (supporting); writing – review and editing (equal). **Inho Kim:** Data curation (supporting); supervision (supporting); writing – review and editing (supporting). **Youngil Koh:** Conceptualization (lead); data curation (lead); funding acquisition (lead); methodology (supporting); project administration (lead); resources (lead); supervision (lead); writing – original draft (equal); writing – review and editing (lead). **Sung‐Soo Yoon:** Conceptualization (lead); data curation (lead); funding acquisition (lead); methodology (supporting); project administration (lead); resources (lead); supervision (lead); writing – original draft (equal); writing – review and editing (lead).

## FUNDING INFORMATION

This research received partial support from Institutional Research Grant number GZ‐2017‐1180 provided by Sanofi. The funders played no role in the design of the study, data collection and analysis, decision to publish, or preparation of the manuscript.

## CONFLICT OF INTEREST STATEMENT

TKK has received a research funding from Nextcure TKK is a consultant for Agenus and Immunobiome. These are not relevant to this manuscript. The authors affirm that the research was carried out without any potential conflict of interest arising from commercial or financial relationships.

## ETHICS STATEMENT

The study was conducted in accordance with the principles of the Declaration of Helsinki and approved by the Institutional Review Board (IRB) of Seoul National University Hospital, and all specimens were collected in accordance with the IRB regulations (IRB Number 1908‐170‐1059, 1705‐031‐852). Written informed consent was obtained from all patients and healthy donors.

## Supporting information


Table S1.
Table S2.
Table S3.
Table S4.
Table S5.

**Figure S1.** Germline variant statistical analysis from high‐depth panel sequencing data. (A) Identified LD germline variants in the MPN patient and normal control cohorts. (B) Frequency of LD germline variants in the MPN total and subtype diseases and the normal control cohort. (C) Comparison of disease prevalence between the MPN total and subtype diseases and the normal cohort using logistic regression modeling (cases with statistical significance highlighted with apostrophe codes). LD, lysosomal dysfunction; MPN, myeloproliferative neoplasm.

**Figure S2.** Information on LD background and marker genes used for cell type identification in bulk RNA and scRNA data sets. (A) Distribution of LD background information across 16 bulk RNA data sets for different disease subtypes in MPN patients. (B) Comparison of expression levels of marker genes used for cell type identification in each cell type of scRNA data. LD, lysosomal dysfunction; MPN, myeloproliferative neoplasm.

**Figure S3.** UMAP distribution for each group in the integrated scRNA data set. (A) UMAP for PV patients with LD background, (B) UMAP for PV patients without LD background, (C) UMAP for healthy donors. LD, lysosomal dysfunction; PV, polycythemia vera; UMAP, Uniform Manifold Approximation and Projection.

**Figure S4.** Differentially expressed cytokine LR genes between PV patients (with and without LD background) and healthy donors. (A) Cytokine LR genes between patients with LD background and healthy donors, (B) cytokine LR genes between patients without LD background and healthy donors. LD, lysosomal dysfunction; LR, ligand–receptor; PV, polycythemia vera.

**Figure S5.** Pseudo‐bulk differential expression of target cytokine LR genes in the target cell type. (A) Comparison between groups with LD background and without LD background, (B) comparison between groups with LD background and healthy donor group, (C) comparison between groups without LD background and healthy donor group. LD, lysosomal dysfunction; LR, ligand–receptor.

Data S1.

## Data Availability

The data that support the findings of this study are available from the corresponding authors upon request.
