# Supplementary figures and images for: Unraveling the impact of lysosomal dysfunction on myeloproliferative neoplasm
Source: Cancer Med. 2024 Sep 25;13(18):e70238. doi: 10.1002/cam4.70238 (PMC11423461; doi:10.1002/cam4.70238)

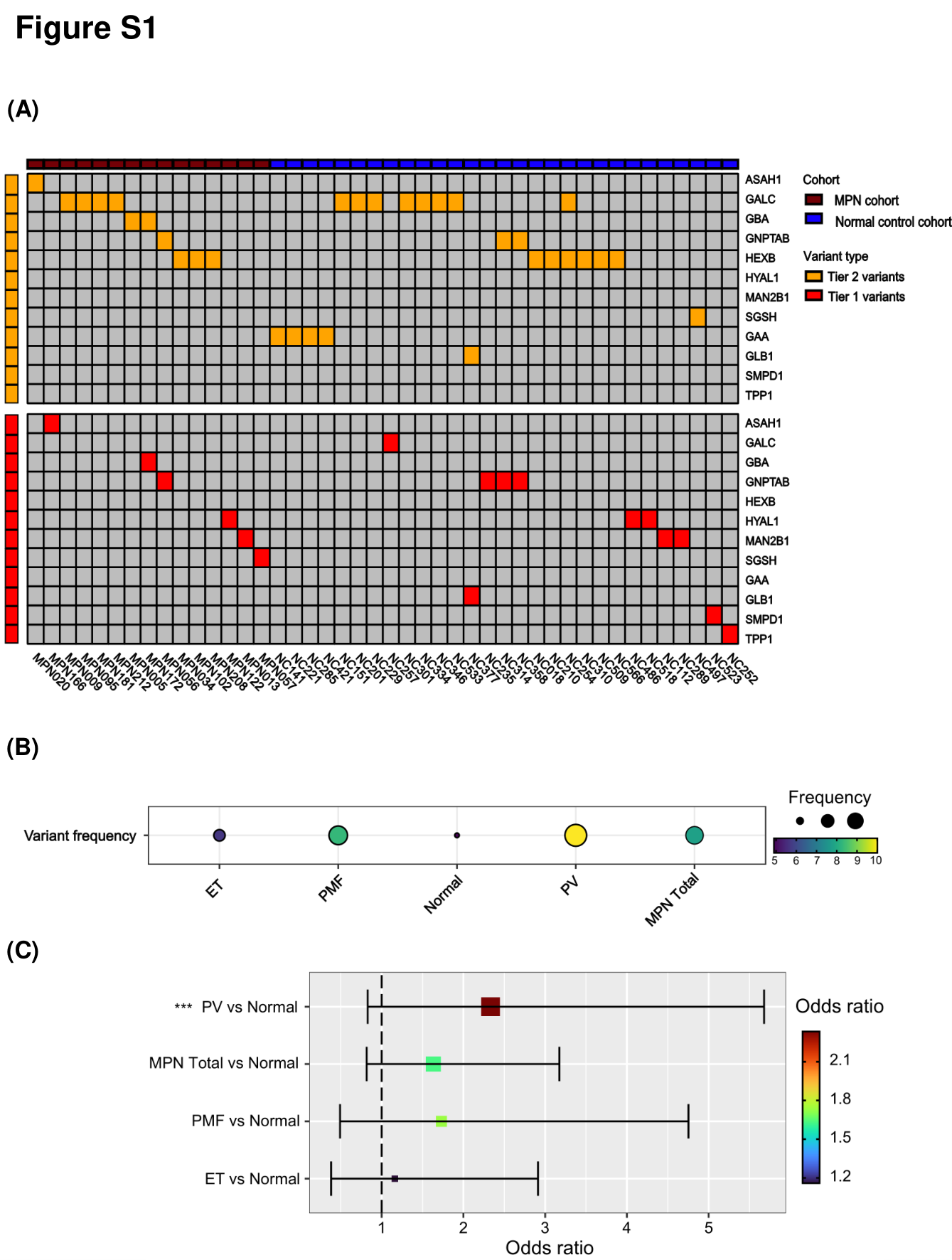

Supplement: Supplementary file 2 — Figure S1. Germline variant statistical analysis from high‐depth panel sequencing data. (A) Identified LD germline variants in the MPN patient and normal control cohorts. (B) Frequency of LD germline variants in the MPN total and subtype diseases and the normal control cohort. (C) Comparison of disease prevalence between the MPN total and subtype diseases and the normal cohort using logistic regression modeling (cases with statistical significance highlighted with apostrophe codes). LD, lysosomal dysfunction; MPN, myeloproliferative neoplasm. [file CAM4-13-e70238-s007.tiff]

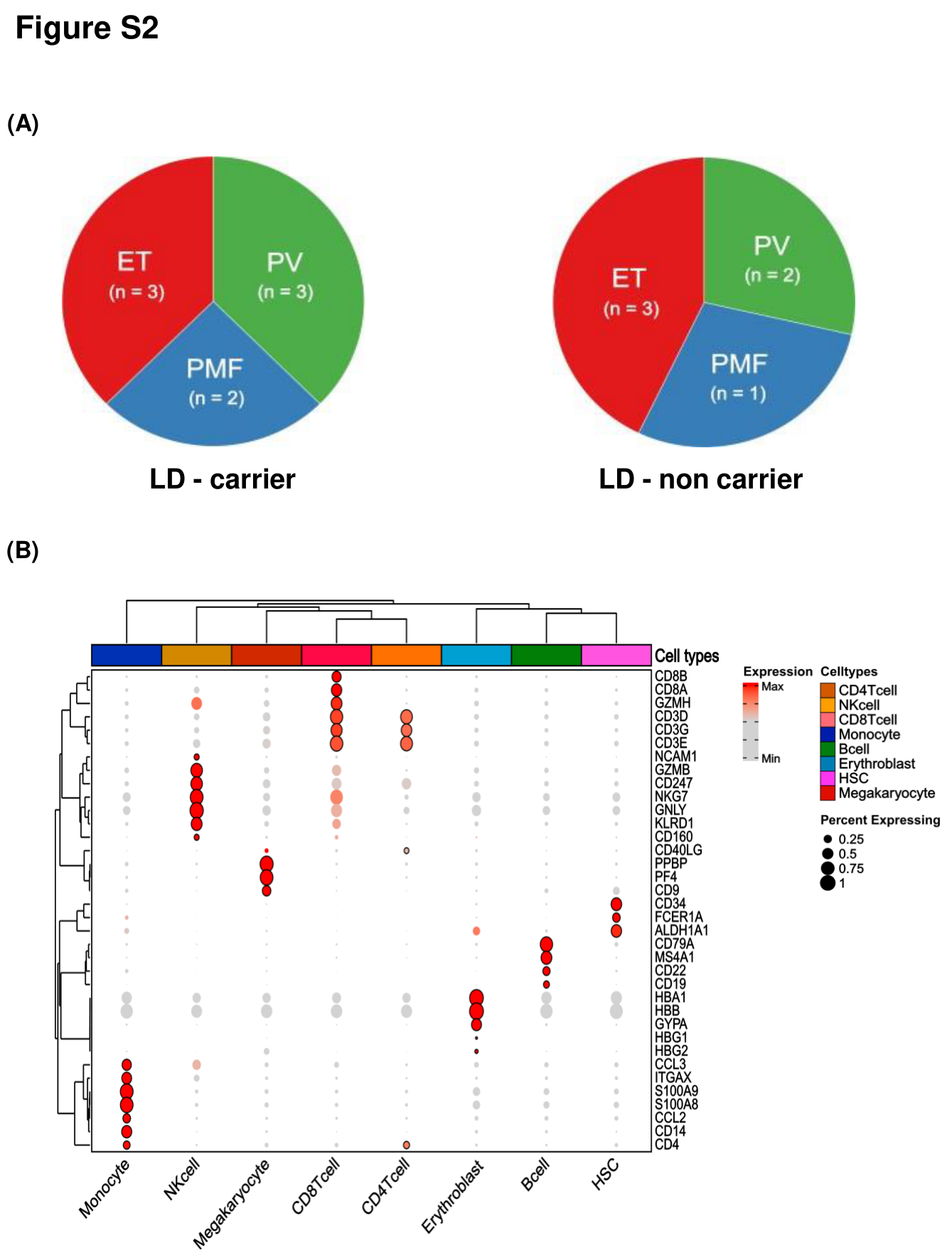

Supplement: Supplementary file 3 — Figure S2. Information on LD background and marker genes used for cell type identification in bulk RNA and scRNA data sets. (A) Distribution of LD background information across 16 bulk RNA data sets for different disease subtypes in MPN patients. (B) Comparison of expression levels of marker genes used for cell type identification in each cell type of scRNA data. LD, lysosomal dysfunction; MPN, myeloproliferative neoplasm. [file CAM4-13-e70238-s005.tiff]

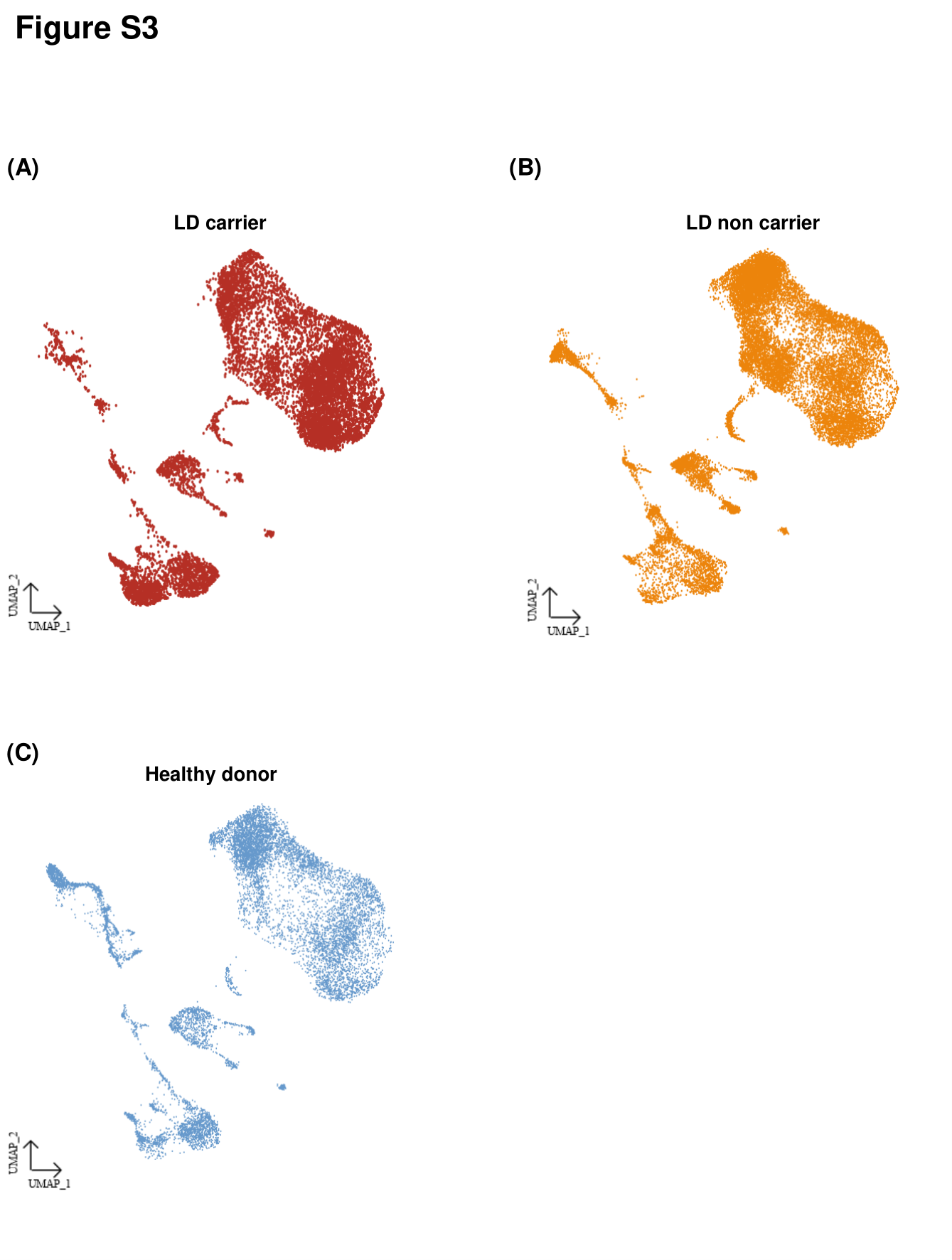

Supplement: Supplementary file 4 — Figure S3. UMAP distribution for each group in the integrated scRNA data set. (A) UMAP for PV patients with LD background, (B) UMAP for PV patients without LD background, (C) UMAP for healthy donors. LD, lysosomal dysfunction; PV, polycythemia vera; UMAP, Uniform Manifold Approximation and Projection. [file CAM4-13-e70238-s004.tiff]

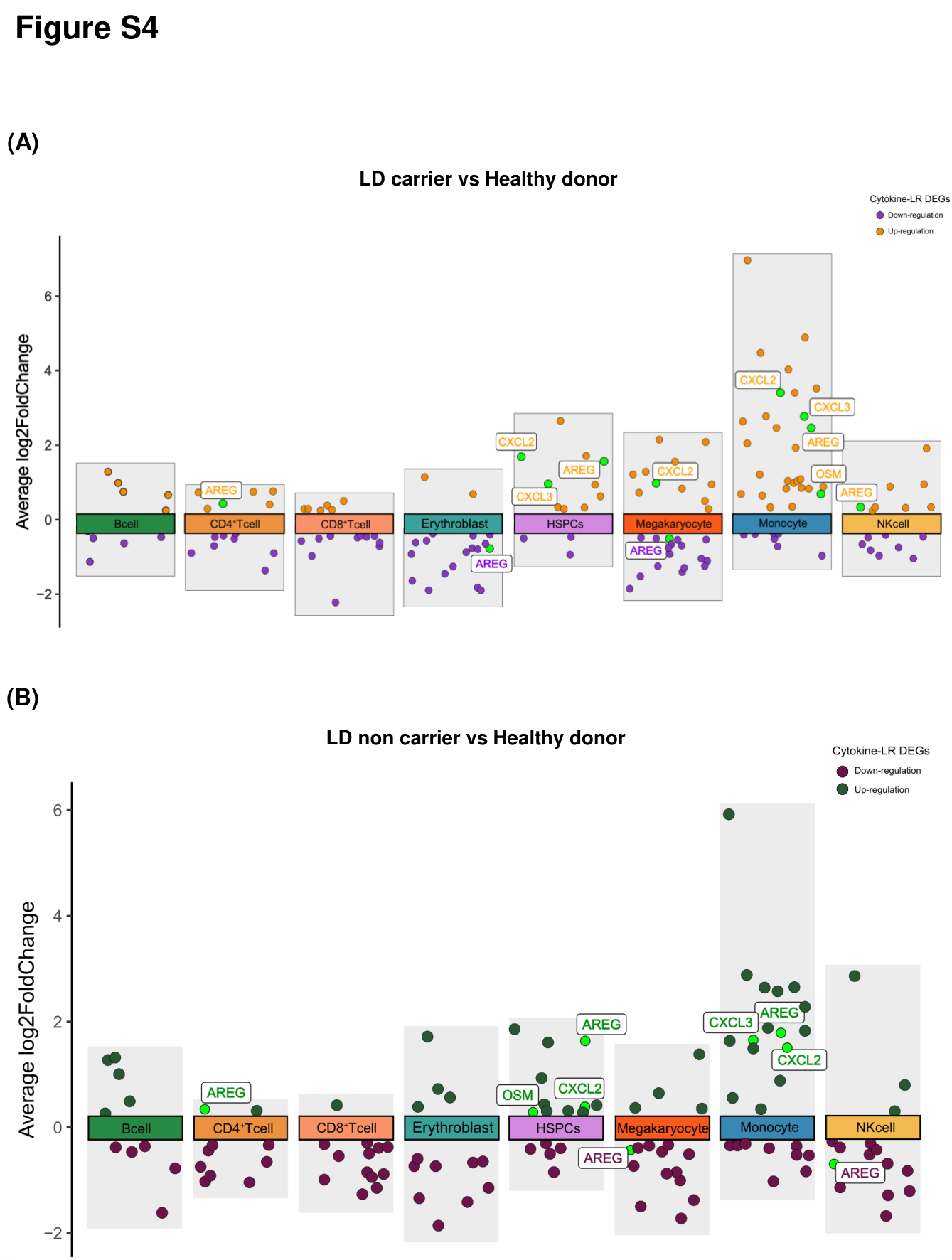

Supplement: Supplementary file 5 — Figure S4. Differentially expressed cytokine LR genes between PV patients (with and without LD background) and healthy donors. (A) Cytokine LR genes between patients with LD background and healthy donors, (B) cytokine LR genes between patients without LD background and healthy donors. LD, lysosomal dysfunction; LR, ligand–receptor; PV, polycythemia vera. [file CAM4-13-e70238-s001.tiff]

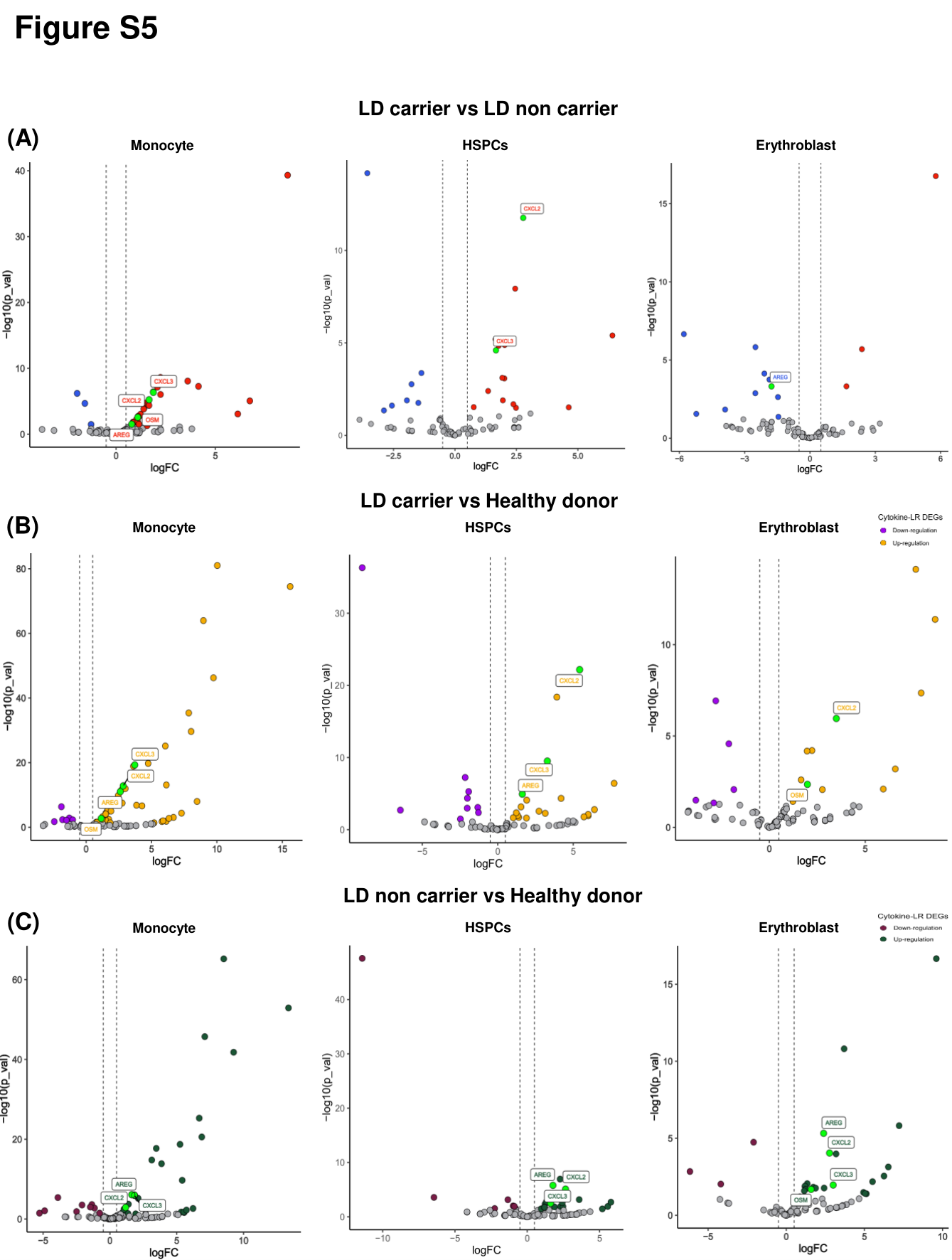

Supplement: Supplementary file 6 — Figure S5. Pseudo‐bulk differential expression of target cytokine LR genes in the target cell type. (A) Comparison between groups with LD background and without LD background, (B) comparison between groups with LD background and healthy donor group, (C) comparison between groups without LD background and healthy donor group. LD, lysosomal dysfunction; LR, ligand–receptor. [file CAM4-13-e70238-s006.tiff]
