# Supplementary material for: Unraveling the impact of lysosomal dysfunction on myeloproliferative neoplasm
Source: Cancer Med. 2024 Sep 25;13(18):e70238. doi: 10.1002/cam4.70238 (PMC11423461; doi:10.1002/cam4.70238)
Supplement: Supplementary file 7 — Data S1. [file CAM4-13-e70238-s003.docx]

**Supplementary Method S1: Genotyping for lysosomal storage dysfunction**

Extracted genomic DNA from peripheral blood collected from patients using the QIAmp DNA Tissue Kit (Qiagen, Hilden, Germany) following the recommended guidelines. DNA from 491 patients in the CFNC cohort was sequenced on the Illumina HiSeq 2500 platform. Libraries were constructed using the ACCEL-NGS 2S DNA Library Kit (Swift Biosciences, Ann Arbor, Michigan, USA), incorporating 42 LD genes (Supplementary Table S1). Subsequently, we identified LD germline variants. The germline variant calling pipeline adhered to the best practices of GATK. Sequence reads were aligned to the human reference genome (hg19) using the Burrows-Wheeler Aligner-MEM algorithm (BWA-MEM). BAM file conversion was performed using Picard. For insertion and deletion realignment, we employed GATK to remove duplicate fragments and recalibrate base quality scores. Single nucleotide variants (SNVs) and indels were detected using the Picard CollectHsMetrics, and germline variant calls were evaluated using GATK's HaplotypeCaller. We performed joint calling by merging variant calls from the VCF files using GATK. Finally, we employed GATK's statistical modeling approach, Variant Quality Score Recalibration (VQSR), for filtering and evaluated sequencing errors. Variants with total coverage less than 10 were excluded, and those with a variant allele frequency (VAF) below 20% were considered biased and excluded. ANNOVAR was used for functional annotation with filter-based annotations to filter and create a consistent set of annotations for the identified variants. Additionally, we utilized the Variant Effect Predictor (VEP) for gene-based information on standard transcripts. To identify rare germline variants specific to the reproductive cell lineage, we performed variant filtering using allele frequency (AF) information from the genomic aggregation database gnomAD. Protein-truncating variants (PTVs) and clinically validated variants were extracted as pathogenic or likely pathogenic variants (PPV) from the ClinVar database and classified into two tiers. Tier 1 variants were defined as PTVs, including splice donor and acceptor sites, frameshift indels, stop gain and loss variants, as well as protein-truncating variants with potential benign or likely benign consequences. Tier 2 variants were clearly defined as genetic variants with ClinVar annotations indicating well-known clinical significance (pathogenic, likely pathogenic, association, and risk factors) and associated phenotypes.

**Supplementary Method S2: Single cell RNA data pre-processing and integration**

The single cell RNA sequencing raw fastq files were aligned to the human reference genome (hg38) using the Cell Ranger pipeline to generate a raw gene-by-cell count matrix. Data quality control was performed to remove sequencing noise generated during single-cell capture. Empty droplets were identified using DropletUtils, and real cell data satisfying FDR < 0.01 were retained. Low-quality cells were removed using the calculateQCMetrics function of Scater based on overall cell quality calculation. Outliers were identified using PCA and removed, and cell-by-cell bias was removed by clustering cells using the quickCluster function of Scran. Cell-specific size factors were calculated using the computeSumFactors function. Gene-by-cell matrix normalization was performed by dividing the number of raw unique molecular identifiers (UMIs) by the cell-specific size factor. Normalized counts were log2 transformed by adding a pseudo count of 1. One thousand highly variable genes (HVGs) for biological variability were selected using the getTopHVGs function of scran. Using the FindNeighbors function of Seurat, a k-nearest neighbor (kNN) graph was calculated based on different principal components (PCs) in each sample, and cell clusters were visualized using uniform manifold approximation and projection (UMAP). An excessive number of red blood cell (RBC) clusters were identified in one sample (MPN 4814) and were removed using Seurat FindAllMarkers function to perform differential expression analysis (DEG) on clusters in which RBC canonical marker genes HBG1 and HBG2 were overexpressed (avg log2FC > 0.25 and p-value < 0.05). To integrate single cell RNA sequencing data generated from different batches, Harmony was used to mitigate batch effects by merging and integrating the data.
